# An interview with Steven J. Lindauer

**DOI:** 10.1590/2177-6709.22.3.026-040.int

**Published:** 2017

**Authors:** Steven J. Lindauer

**Affiliations:** » Norborne Muir Professor and Chair, Department of Orthodontics, School of Dentistry, Virginia Commonwealth University (VCU).

## Abstract

There are so many compliments to Dr. Steven Lindauer that is hard for one to figure where to start from… Well, travelling backwards in time, all the way to the year 2000, I went to Virginia to study English at Virginia Commonwealth University (VCU), in Richmond, Virginia, USA. During my daily walks to the English school, I used to pass by the School of Dentistry, where the Department of Orthodontics was. That was the place where my very first “contact” with the VCU happened. In 2015, 15 years later, I had the pleasure to go back to the VCU and spend two days with Dr. Steven Lindauer. I have to confess that I was anxious and nervous to get to know not only the Chair of the Department, but also the Editor-in-chief of “The Angle Orthodontist”. Since the very first moment I could experience how incredibly positive and pro-active the environment within the Department was. Staff members, Residents and Faculty members used to work very gladly and in perfect synergy. After a quick chat with the residents, I heard from them: “Dr. Lindauer is an unprecedented human being! Besides a brilliant Professor, Researcher and Administrator, he is like a father to all of us!” Besides this touching testimony, I also heard flattering compliments from workmates to the great friend, leader and partner Dr. Lindauer was. Here goes some food for thought, though. Unfortunately, the leader is often compelled to resort to unpopular measures in order to enforce compliance. Dr Lindauer is the living proof that a boss, a leader, can be a light, humble, friendly and highly charismatic human being. In 2016, I was given a second chance to enjoy Dr. Lindauer’s company when he visited Brazil as a guest lecturer in Salvador, at the Federal University of Bahia and the Brazilian Association of Orthodontics (Bahia Chapter). I noticed, once again, that besides an excellent lecturer and careful clinician, he mastered orthodontic mechanics very proficiently. And needless to say at length about his brilliance ahead of “The Angle Orthodontist”... It didn’t take me long to realize that his virtues by far exceeded the boundaries of the professional domain. Despite his utterly busy schedule, he is still able to dedicate time to his parents, taking them to trips around the world. Interestingly, at every international trip, he always remembers his puppies (Memphis, Baxter and Kingston - in memoriam), taking sightseeing pictures to immediately Photoshop them into. Having done the well deserved introductions to our distinguished interviewee, I would like to offer righteous acknowledgements to the colleagues Jorge Faber, David Turpin, Bhavna Shroff and David Normando, for having accepted the invitation to take part in this interview. I also would like to offer my heartfelt thanks to Dental Press for having entrusted me with the honor to conduct this project. I wish all readers as delightful and rich of an experience when going through this interview as it’s been the scientific path that brought us all here in the first place. No doubts, you stand in face of a life dedicated to Orthodontics. (Andre Wilson Machado, interview coordinator)

## Abstract (pt)

São tantos os elogios ao Dr. Steven Lindauer que nem sei por onde começar… Bem, voltando ao passado, no ano de 2000, fui estudar inglês na *Virginia Commonwealth University* (VCU), em Richmond, Virginia, EUA. Durante minhas caminhadas diárias à escola de inglês, passava em frente à Faculdade de Odontologia, onde se localizava o Departamento de Ortodontia. Ali se deu meu primeiro “contato” com a VCU. Quinze anos depois, em 2015, tive o prazer de retornar à VCU e conviver com o Dr. Steven Lindauer durante dois dias. Confesso que estava ansioso e nervoso para conhecer o não apenas *chairman* do departamento, mas também *editor-in-chief* da *The Angle Orthodontist*. Desde o primeiro momento, pude perceber como o clima do departamento era positivo e altamente pró-ativo. Funcionários, residentes e professores trabalhando em total sinergia e alegria. Em um momento de bate-papo com os residentes, ouvi deles: *“Dr. Lindauer é um ser humano sem igual! Além de brilhante professor, pesquisador e administrador, é um pai para todos nós! Ele cuida e briga por nós!”* Além desse lindo relato, ainda pude ouvir de seus colegas de trabalho elogios contundentes ao grande amigo, líder e parceiro. Aqui, vale uma reflexão: infelizmente, muitas vezes, o líder para impor respeito precisa usar artifícios antipáticos; porém, o Dr. Lindauer é a prova viva de que um chefe, um líder, pode ser humano, leve, humilde, simpático e altamente carismático. Uma segunda chance de desfrutar da convivência com o Dr. Lindauer veio em 2016, quando, em visita ao Brasil, esteve em Salvador ministrando um curso a convite da Universidade Federal da Bahia e da Associação Brasileira de Ortodontia (seção Bahia). Mais uma vez pude perceber, além do excelente palestrante e cuidadoso clínico, o seu minucioso domínio da mecânica ortodôntica. Nem julgo necessário alongar-me a respeito do brilhantismo à frente do *The Angle Orthodontist*... Não demorei para perceber que suas qualidades ultrapassam a esfera profissional. Embora possua uma agenda lotada, ainda consegue espaço para se dedicar aos pais e levá-los a viagens pelo mundo afora. Curiosamente, em cada viagem internacional, sempre se lembra de seus cães (Memphis e Kingston -*in memoriam*), registrando fotos turísticas para, em seguida, inseri-los nelas via Photoshop. Feitas as devidas apresentações do nosso ilustre entrevistado, dedico meus cordiais agradecimentos aos colegas Jorge Faber, David Turpin, Bhavna Shroff e David Normando, por aceitarem o convite de participar ativamente dessa entrevista. Agradeço, ainda, à Dental Press, pela honra a mim concedida, em conduzir essa experiência. Desejo a todos os leitores que essa leitura seja tão prazerosa e rica quanto o trajeto científico que nos trouxe a essa entrevista. Sem dúvida, vocês estão diante de uma vida dedicada à Ortodontia. (Andre Wilson Machado, coordenador da entrevista)

**Figure:**
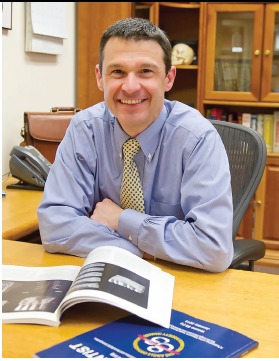

## Could you please provide us some of your dental/ortho background? Andre W. Machado

I decided that I wanted to be an orthodontist when I was 12 years old, during my own orthodontic treatment. I liked my orthodontist. He was happy and friendly and he really seemed to enjoy what he was doing. I appreciated that orthodontic treatment was one of the first “doctor experiences” that I had where I could attend the appointments without my parents accompanying me and the staff and doctor treated me like a real person. The doctor’s job didn’t look hard and I thought it would be a fun job. Also, I saw there were opportunities to fill my office with games and distractions that would appeal to 12 years olds. This seemed like a brilliant idea at the time.

Around that same time, I actually wrote to the American Association of Orthodontists and asked for information on how to become an orthodontist. There were no computers then, so the only real source of information would have been going to the library or writing to an organization asking for information. They sent me material about requirements and a list of schools in the US with brief descriptions of the orthodontic program offered by each. That’s when I learned that I would have to take a lot of math and science courses to get into dental school and that I would actually have to go to 4 years of college, followed by 4 years of dental school, just to start an orthodontic education. Most of the programs were 2 years long but some were 3 years and I actually remember thinking, even when I was 12 or 13 years old, that it would be better for me to attend a 3 year program so I could learn the most I could and not rush through it.

From then on, whenever any adult asked “What do you want to be when you grow up?”, I would answer that I wanted to be an orthodontist, and that seemed to impress them. The math and science requirements fit my talents anyway so it didn’t worry me. I guess I am stubborn because I didn’t change my goal of becoming an orthodontist. I always enjoyed the math and science courses.

I chose to go to college at the University of Pennsylvania because I figured it was the best college that also had a dental school (also Harvard is a great college that has a dental school but I was not accepted at Harvard). Ironically, I did not go to dental school at The University of Pennsylvania because it seemed too expensive and, luckily, I chose to attend dental school at The University of Connecticut. It was only about an hour and a half’s drive from my parent’s house. Dental school was probably the hardest I had ever worked in my life. We had class 6 days per week with only Sunday off and the dental and medical students took classes together for the first two years. We learned all the medical course until then and then did all of our clinical dentistry in the third and fourth years. Many of the dental students in my class had to repeat at least one year because they failed. It was very tough.

I knew that it was very competitive to get a spot in an orthodontic program after dental school. I had done well in dental school, had gotten top scores on the National Board exams, and also had won an award for the research I had done on TMJ function. But, still, there was uncertainty. Orthodontics seemed like the perfect fit for me because it seemed like the most logical and scientific of all the specialties. The idea of applying physics to biology was especially interesting to me and, without really knowing that I had hit on the exact right answer, that was what I told Dr. Burstone during my interview for entry into his orthodontic department at Connecticut. I also remember saying that I wanted to open a private practice of orthodontics, teach at a school part-time and do research, and be editor of an orthodontic journal. Dr. Ravi Nanda, who was also on the faculty in the orthodontic department at Connecticut told me during the interview that doing all of this would be impossible. I never imagined that I would end up teaching full-time at a university, but I think Dr. Burstone knew even then that I would.

The University of Connecticut had a 2 year and 3 year option for the orthodontic program at that time and, true to my stubbornness, I chose the 3 year option so I could learn more and also get a Master’s Degree. I thought about a PhD but the program was a minimum of 6 years and that seemed too long. I loved learning biomechanics and treating patients in the clinic. I did research outside of the orthodontic department, with Dr. Thomas Gay, and did a study on EMG activity related to craniofacial morphology. I got a US government scholarship that paid for my tuition and gave me money to live during my orthodontic program. In return, I agreed that I would teach for one year after I graduated.

I got job offers at 3 programs to teach after I graduated. Dr. Sam Weinstein, who was also on the Connecticut faculty, had arranged for me to interview at the Medical College of Virginia (now VCU) with Dr. Robert Isaacson. I went to interview in Richmond, Virginia and it was so exciting there because Dr. Isaacson had so many logical and creative ideas, that I came back to Connecticut and told everyone I was moving to Richmond. And I did.

When I started working at VCU, I intended to do research in the area of muscle function. I bought some equipment and wrote grant applications but the topic of muscle function was not popular enough to obtain NIH funding. Eventually, I had to abandon the idea. Meanwhile, Dr. Isaacson had become interested in biomechanics and I knew a lot about biomechanics from my time in Connecticut. We were an ideal team. He and I approached biomechanics from different viewpoints. He approached it as a clinician learning biomechanics with clinical orthodontics as the framework and I approached it as a student would, theoretically and ideally. Together, it was really perfect. We wrote a lot of papers together and I also had an NIH grant funded to study 3D biomechanics. That was the topic of my first talk at the AAO annual session, which happened to be in Denver that year. My lecture was sandwiched in between Dr. Burstone and Dr. Thomas Mulligan and there were about 2000 people in the room. I was nervous but it went very well and it was exciting. After that, I also had some more biomechanics projects funded by the AAO Foundation.

Dr. Isaacson and I worked together on the faculty at VCU for 13 years. We had some other excellent colleagues also during that time: Dr. Loretta Rubenstein, Dr. Joe Rebellatto, Dr. Moshe Davidovitch, and Dr. Rose Sheats, but each of them only stayed a few years and then moved on. Dr. Isaacson and I were there together for the whole time. Then, one day, Dr. Isaacson had a disagreement with the Dean of the school and he stepped down as Chairman. That was in 2000 and I became Chair of the department. I was 39 years old when I became Chair. Dr. Isaacson stayed to help me with the transition for a year, and then he retired and moved to Minnesota to be near his family. For over a year, I was the only faculty member in the orthodontic department at VCU. I learned a lot during that time. I learned that the only way to run a department effectively by myself was to make sure that everyone knew it was important to contribute to the success of the department: the residents, the part-time faculty, and the staff. Everyone had to take responsibility for making the department work in the best way that it could. It was a crisis in a way, trying to run everything myself, and it brought everyone together. I will never forget that experience.

By the end of 2002, I had hired two new faculty members to work with me at VCU: Dr. Eser Tufekci who was graduating from the orthodontic residency at the University of Minnesota, and Dr. Bhavna Shroff, who was on the orthodontic faculty at the University of Maryland and who had graduated with me in my orthodontic class from the University of Connecticut. I was the biomechanics expert, Dr. Tufekci had a PhD in biomaterials, and Dr. Shroff was known for her work in biology of tooth movement. It seemed like a perfect team.

## What is the approach that you and your faculty take in educating new orthodontists at VCU? In other words, what is the VCU’s teaching philosophy? Andre W. Machado

The teaching philosophy that we have at VCU is that the students/residents learn best when the material is clinically applicable. In other words, we try to get them involved with clinical practice as soon as possible so they can apply what they learn immediately, so the material is retained better. In the US, we call this “learning to swim by being thrown into the pool.” Of course, that is really not possible when patients are involved.

In actuality, what we do is give the new residents an intensive 4 week academic introduction at the start of the graduate program and then get them involved with patient treatment directly. My experience tells me that they actually forget many of the basic pieces of information we introduce during those initial few weeks until they are able to apply the information clinically. However, once they have started treating patients, they retain the information much more reliably.

Another key element in our teaching philosophy is the principle of participatory learning. There are very few instances where the faculty spend time lecturing to the residents. Instead, we ask the residents to present and discuss material and the faculty are there to supervise, intervene, and stimulate discussion. By motivating the residents to actively participate in the learning and teaching process, I feel that they take on a feeling of greater responsibility, actually end up learning and retaining more, and develop a sense of a need for continued learning throughout their career.

## I know you have had the opportunity to travel a lot. What differences do you see in the profession across the different countries that you have visited? Andre W. Machado

One of the greatest benefits of working in an academic environment and also being Editor of an orthodontic journal is the experience I have been able to have traveling to different countries and seeing how orthodontics is taught and practiced differently in different areas. Honestly, there is less difference than I would have anticipated initially. The more I have learned, the more I think that there are more similarities than differences internationally.

There are differences in the academic model around the world. In the US, students attend post-secondary school (college) and must graduate before completing 4 years of dental school. Then, continuing on to orthodontic specialty training can be as little as 2 more years. Many other parts of the world require completion of secondary school (what we in the US consider “high school”), and then dental school is an additional 5-6 years followed by at least 3 years of orthodontic specialization.

At one time, many parts of the world have looked to the US as the leader in the specialty of orthodontics. Since Edward Angle significantly shaped the direction of orthodontics in the early part of the 20th century, I don’t think this is unexpected. However, the world is a much smaller place now. Many faculty members from all over the world have been educated in the US and Europe, and the general globalization of thoughts and ideas have made what we learn, teach, and practice, much more homogenous around the world.

## I had a chance to spend some time at VCU and share with your residents and Faculty. At that time I felt how your residents respect and admire you. In addition, everybody seemed to work so happy. This is somehow challenging because you are in charge. How do you manage to be a Chair and make your team love you? Andre W. Machado

One of the things that I think is unique about the environment we have been able to create in the orthodontic program at Virginia Commonwealth University is a sense of shared responsibility and ownership. I have to attribute most of this to the previous Chair and one of my important mentors, Dr. Bob Isaacson. He was a person who always operated everything very democratically and instilled in all of the junior faculty and residents alike that they shared responsibility for the success of our orthodontic program.

For me, one of the most revealing moments occurred, ironically, when Dr. Isaacson decided that it was time for him to leave and retire. At that time, his departure caused a kind of panic around the orthodontic department at VCU. It was in 2000, and he and the Dean of the Dental School had a disagreement. He came to me after their meeting and disclosed that he was going to submit his resignation the following day. I was quite scared and was willing to do whatever he wanted to resolve this situation, even if it meant quitting my job and moving with him to a new job. He and I, at that time, had been working together quite successfully for 13 years. But his plan was to retire and move to Minnesota and I, of course, being the Program Director, but relatively junior in status, was not ready to retire. Instead, I accepted the Dean’s offer to become the new Chair and take on the responsibility of moving the department forward.

By 2001, Dr. Isaacson had left, and the other junior faculty member in the department left for private practice leaving me to run the department with no other full-time faculty members. It was at that moment that I realized that the department could not survive unless the residents, part-time faculty members, and even the staff took on roles of responsibility to ensure the department’s future success. It was then that I learned that empowering the participants (residents, staff, and part-time faculty) to do what was right for the future of the department was actually more powerful than having one person (myself) directing all of the actions.

Since then, I have successfully retained full-time faculty members, Drs. Bhavna Shroff and Eser Tufekci, who bring unique talents to our VCU Department of Orthodontics. However, it is the shared responsibility of ALL of the participants including the residents, part-time faculty, and even the staff, that makes the department function at a higher level overall.

Of course, you can imagine that it does not always work ideally. There is a constant need to remind everyone of their role in making the didactic, clinical, and overall morale of the department continue to work at the optimum level.

## You have a strong background with Biomechanics. What are the indications that the segmented arch technique provides real advantages compared to continuous arch technique? Andre W. Machado

Segmented mechanics is, contrary perhaps to common belief, the most simple way to understand what we are really doing when we perform orthodontic treatment. Segmented mechanics breaks down the complex force systems we develop in modern fixed appliance treatment, into only two components and therefore it is more simple to teach and understand. Rather than jumping forward and trying to decipher the complex systems developed when we are dealing with all of the teeth in the dental arch at one time, segmented mechanics joins the teeth into discrete units to simplify the analysis of force systems so they are only applied on two units at a time.

This way of thinking is not only applicable to the fixed edgewise mechanics that are the most commonly used at the present time. The same way of thinking also applies to new developments such as aligner therapy. It is overwhelming to try to analyze the complex systems that are developed when an entire arch is considered. However, if you can join the tooth units together into discrete, larger, groups, then it is simpler to understand the favorable and unfavorable tooth movements that will occur when even basic alignment is attempted.

I think the key is to be able to recognize the geometries of bracket alignment (or tooth alignment in the case of aligner therapy) that will create either favorable, or unfavorable, effects when routine treatment is performed. If the geometry is favorable, and especially if the patient exhibits favorable diagnostic and growth potential traits, then treatment is likely to proceed favorably no matter what you do. However, if the geometries are unfavorable, meaning they are going to produce unwanted side effects in any dimension, and especially if the patient exhibit unfavorable facial or growth tendencies, then THOSE are the situations that require additional biomechanical care and knowledge. Recognition of which are favorable and which are unfavorable situations is the key to successful diagnosis and treatment of orthodontic patients. When the situation is unfavorable, treating the patient using segmented arch principles allows you to control the negative side effects more predictably.

## Ethical misconducts in science have been a big concern in all fields. What are the most common ethical issues in manuscripts submitted to The Angle Orthodontist and how do you handle them? Jorge Faber

Scientific misconduct is something I have seen much more often than I ever imagined I would when I first became Editor of The Angle Orthodontist. I always knew that it was possible that researchers could commit fraud by making up data rather than conducting honest experiments, but I did not imagine that this would happen in the orthodontic literature. My thinking was that orthodontists are primarily clinicians and that the pressure to publish was not as great as it would be in other academic fields such as basic science or the humanities. However, at this point, I believe anything is possible.

Most of the conduct violations I see commonly, I believe, are honest mistakes, misunderstandings or, at worst, an attempt to accelerate the otherwise tedious process of having papers accepted for publication. Oftentimes, there are requirements placed on graduate students or junior faculty to have papers published and they try to do this by stretching the ethical rules that govern scientific publishing.

The most common violation I see is from authors who copy sentences or summaries from other sources (from other published paper or actually anywhere on the internet) and insert them into their papers as background information or discussion points. Clearly, this is a copyright violation but I believe for the most part, that the authors just see it as an easy way to communicate known information without putting too much effort into the thought process. Alternatively, it is a way for authors who are not fluent in English to communicate their own general thoughts to the readers without having to formulate the words, sentence structure, and grammatical details into perfect language for publication. Clearly, it is an added burden for non-English speaking authors to write fluently in English to have their papers accepted. Unfortunately, copying other people’s words is plagiarism and it is not ethical to do this. Editors from most of the scientific journals, including The Angle Orthodontist, have access to software that identifies these violations. When this is identified, the author is notified that the paper is similar to other material and the paper is rejected.

Another very common violation I have encountered is when authors try to speed up the publication process by submitting their paper simultaneously to more than one journal for consideration. Every journal prohibits this practice and has the authors sign a statement promising that the paper will be submitted only to one journal at a time. Generally, the author is caught because orthodontic journals use many of the same reviewers and the reviewers themselves recognize and report that they have been asked to review the same paper simultaneously by more than one journal. When this happens, once again the paper is rejected, usually by all of the journals involved, and the authors may be punished by being banned from being able to publish in those journals for a specified amount of time.

Unfortunately, I have seen more blatant violations including authors submitting someone else’s data for publication (caught by reviewers familiar with the original work), new authors submitting someone else’s paper with new data (also caught by reviewers), and authors submitting multiple versions of papers they have already had accepted for publication in other journals (caught by readers of the journal). In more than one case, The Angle Orthodontist has been forced to retract papers that were already accepted for publication because a violation came to our attention after the paper appeared online. In one recent case, all of the co-authors were banned from future publications in more than one orthodontic journal for the next 3 to 5 years.

In the past, journal publications were available only in print and it was difficult to assess the number of duplicated and copied work from journal to journal. In today’s digital world, the information is easy to access and it is much more difficult for authors to get away with plagiarism and publishing the same work in multiple journals. Authors may be unaware that their papers are sent to reviewers who are experts on the specific topic of their paper and it is not unlikely that multiple journals will use the same small number of experts familiar with that topic. Therefore, it is hard to get away with cheating the system.

On an even more serious note, I am aware of instances in other fields in which authors have been found guilty of actually fabricating data to prove their hypothesis. High powered statistical analysis can be used to prove that the data presented in a paper is so unlikely from a statistical viewpoint, that the results are, by all statistical probability, fictitious. In these cases, the authors’ careers are terminated. I am not aware of this happening in orthodontics, yet.

## We have less randomized clinical trials in our field than we would like to have. Having this as a background - at this specific point in time, and considering the topics that you have been directly involved with in your own research work - what would be the RCT study that you would love to read and why? Jorge Faber, David Normando

Randomized clinical trials are considered to be the gold standard of proof to show the comparison between two or more clinical approaches to any condition. On the other hand, the argument is made that randomization creates an artificial environment in which all of the patients are considered to be equal at the start, when experienced clinicians know that is not true and might have approached treatment differently in certain patients depending on some of their individual characteristics. Despite this drawback, I am a great proponent of the randomized prospective approach to clinical decision-making.

Superimposed on the problem of human variation which is always a drawback of the blind randomization method, are the ethical concerns we have for treating patients in different ways or for having a control group that remains untreated while we measure treatment effects. Some of the measurement techniques employed in orthodontics today, in the past meaning multiple cephalometric radiographic exposures but not meaning multiple CBCT exposures, also raise ethical concerns. It is difficult to get studies approved and then, papers published, when these ethical boundaries are breached.

Recently, I heard a lecture by Dr. Bill Proffit in which he discussed the way in which we conduct and report clinical trials in the orthodontic literature. I think he has some good points. If we accept that there is a large amount of biological, individual human variation for which we can not account, then it is probably not appropriate to report clinical outcomes as a mean with the variation around it. Instead, we should be considering the proportion (or percent) of patients who improved, stayed the same, or became worse from the treatment modality we are studying. On a basic level, that would give us data to inform our patients of the probabilities that we will make them better if we employ a particular treatment strategy. As a next step, we can further explore what characteristics would make a specific patient more or less likely to experience an improvement or detriment from that particular treatment.

So, I have avoided the initial question. Undoubtedly, we would all agree that some patients derive benefit from early treatment, some patients derive benefit from functional appliances, some patients remain stable with arch expansion, etc. I would like to know how we can predict WHICH are the right patients to treat with these protocols. Honestly, I am not sure that the current methods we are using to conduct randomized clinical trials will lead to these answers directly.

## Many 3D studies have been published in recent years as a result of computer science advances. How do you believe these technologies will change orthodontic clinical practice? Jorge Faber

The 3D technologies we have to examine the changes that occur in our patients have already had great impact on the way we treat patients. The most notable example, I believe is in canine impaction patients. Now, with pre-treatment CBCT diagnosis, we can map out the most favorable route to move an impacted canine to avoid interference with other structures or other teeth to cause iatrogenic damage. Alternatively, it can alert us to situations when a different mode of treatment should be considered altogether, such as perhaps abandoning our original plan to retain the impacted tooth or adjacent tooth.

In research, CBCT has allowed us to view the impact of treatment on alveolar bone when we move teeth in much more detail than we knew previously. Using only 2D projections, it was nearly impossible to assess changes in buccal or lingual bone support of teeth. Now, we can assess whether different types of tooth movement - tipping versus translation, for example - can result in better or worse bone support outcomes for specific teeth during treatment.

On the other hand, the first impulse for most clinicians was that CBCT would give us a better and more complete method of facial and skeletal analysis of our patients for diagnostic and treatment planning purposes. In other words, the thought was that we would develop a three-dimensional cephalometric analysis that would answer a lot of the unanswered questions and uncertainties that we had in making orthodontic treatment decisions. That has not occurred. Perhaps it is a limitation of our own minds to be able to comprehend 3D analysis and use it effectively. Authors continue to send in proposed analyses as papers to be considered for publication. Up to this point, however, nothing revolutionary in this particular aspect has really risen to the level that we may have expected when CBCT was first introduced.

## Regarding the submission of either basic or clinical research articles over the past 10-15 years, has there been a change in what you accept for publication? David Turpin

There has been dramatic change in the type of articles accepted for publication today compared to 10 or 15 years ago in The Angle Orthodontist and, I imagine, in other international orthodontic journals. We actually conducted a study looking at this question and tracked definite changes.

The most obvious and general shift in the journal has been toward scientific research papers and away from opinion, general review, and case presentations. Even 15 years ago, the focus of the journal was on scientific research but, now, that is really the only type of paper we consider, along with a few selected case reports.

In addition, the type of research has changed as well. I think this should be expected as the specialty of orthodontics evolves over time and clinicians as well as academic orthodontists are interested in different topics than they were 10 or 15 years ago. Generally, current topics such as accelerated orthodontic techniques, new digital applications, aligner treatment, and psychological and quality of life impacts of treatment occupy a greater proportion of the journal now than cephalometric comparisons among population groups, retrospective case analyses, and bonding studies that were popular before. Reviewers are much more critical of retrospective studies than in the past and are often demanding that research be planned prospectively.

## Are you pleased with this balance? If not, how would you like to see it change? David Turpin

There is a balance of clinical and basic research in The Angle Orthodontist. I have not calculated the percentages and I guess that the dividing line between clinical and basic is really not that clear. Most people would consider a study of two different treatment techniques looking at speed of tooth movement to be a clinical study but, if it were conducted in rats, maybe they wouldn’t see the clinical application as clearly. Both of these kinds of studies are important to furthering our knowledge about the procedures and their future potential. I would estimate that 70% of the papers in the journal would be considered clinical and I think that is a good balance. 

When papers are reviewed and I get the recommendations back, I need to make a decision of whether or not to push the paper forward and publish it, or to tell the authors that they should send it somewhere else. About two-thirds of the time, the decision is not too difficult because the reviewers send a relatively clear message one way or the other. But, the other times, I have to decide based on the topic of the paper, whether it is something I think the readers want to see, or whether the paper would be more appropriate for a different audience. Since The Angle Orthodontist is clearly considered as “a clinical journal,” I would tend to recommend that authors send their papers elsewhere if I don’t perceive a clear clinical application.

## Have you developed a way to evaluate the quality of reviews received, and if so, have you found it to be productive? In other words, how satisfied are you with the general quality of the reviewing effort? David Turpin

There are definitely differences among reviewers in the quality of the reviews they produce. In addition to quality, there is a reliability and efficiency consideration. Those latter quantities are easier to evaluate. The Editorial software we use routinely reports on how frequently the reviewer agrees or declines to do a review, how often they agree but don’t actually deliver (which is really the worst possible outcome), and how long on average they take to complete a review. We give our reviewers 28 days to return their evaluations and most reviewers actually meet that deadline successfully or exceed it by only a few days at worst.

Usually what holds up my decision when it is delayed is an inability to get reviewers to agree to take on the review for a particular paper. It may be on a topic they have low interest in or they may perceive just from the title or abstract that the paper won’t be exciting to read. I find that authors often don’t spend enough time constructing the right title or being careful writing the abstract. They may not realize that this is the first piece of information the reviewers receive on a paper and, therefore, it is arguably the most important part. If there are typographical errors in the title or abstract, the reviewer is immediately turned off.

I left the question regarding the actual quality of the reviews we receive to answer last. The editorial software does provide a way of maintaining data on the quality of evaluations provided by individual reviewers. It would be a subjective rating assigned by the editor or assistant editor. As of yet, I have not really made use of this function. Each paper gets sent out to 3 reviewers and we usually receive at least 2 evaluations back. Generally, but not always, I feel the reviews are helpful and I feel confident in my decision. Otherwise, I read the paper myself, have a colleague read it, and sometimes also send it out for further review. Also, over time, I feel the general quality of the reviews I receive is better now than in the past because I have learned which reviewers will not send detailed thoughtful reviews and I don’t send papers to them anymore. Sometimes, actually quite rarely, authors complain that one of their reviewers misinterpreted something in the paper in error, but many times it is a combination of poor clarity in the paper as well as inattentive reading on the part of the reviewer.

## It’s obvious that the publication of ‘print journals’ is nearing an end. What technological methods can be used to encourage more exposure to current research findings being produced worldwide? Are you able to take advantage of these methods of communication? David Turpin

I agree that there will come time soon when journals will no longer be published in print. Actually, every time the Board that governs The Angle Orthodontist meets, we talk about this issue. Ironically, even though the online circulation and readership of the journal is more than 30 times the print subscription circulation, authors and advertisers show a strong preference for the print version. At this point in time, having the journal come out in print seems to boost the credibility of the journal compared to online only.

Currently, the online version of the journal is treated in the same way as the print version. Once it is published, there is no way to change it except by publishing an erratum in a later issue. This is a requirement of the indexing engines. In a couple of cases, we have completely removed articles that were posted as “online early” due to some misconduct issues but this would not be permissible if the paper had appeared in an actual online issue.

All of this is related to journal stature and credibility. When we do, someday, give up the print copy and become online only, I would envision The Angle Orthodontist as a peer reviewed, indexed journal, that just happens to be published online. I don’t think I would want it to be interactive or subject to online postings without censure about particular articles. Controlling the quality of what gets published and posted is important.

## How do you manage the opportunities to interact with your readers more directly following the publication of controversial research findings? Do you value letters-to-the-Editor, or activate a blog to speed-up the interaction? David Turpin

When Letters to the Editor come in, I read them and decide if they raise an intellectually interesting issue or whether the author is just trying to get attention. Oftentimes, I feel that the author of letter has an opinion they want to share but it has no evidence or research behind it. In some cases, I will suggest to the author of the letter that they conduct a research project to show what they are trying to hypothesize.

If they are valid points and worthy of discussion, I usually edit the letter to make it shorter and more to the point and we send it to the authors of the original paper for comment. Then, the letter and the author’s comments are published together in the next available issue.

Overall, I don’t feel that The Angle Orthodontist gets a lot of letters that are worthy of discussion and publication. In terms of time, I spend a disproportionate amount of time on the few letters we get in comparison to the 1000 article submissions that come annually. When a letter comes in, I have to fact check it first and make sure the author’s points are all valid and noteworthy as well as relevant to the article. There is no outside review system for Letters to the Editor. Honestly, I would rather spend my time on the articles themselves than on letters that readers have written about the articles. I’m actually quite short of time overall.

## Will you publish research that has been funded by a large corporation if fully acknowledged near the beginning of the article? To be more specific, where do you draw the line? David Turpin

We have published some articles written by authors with disclosures regarding company affiliations. Also, we have rejected articles where it was clear that the authors had tried to bias the wording in papers where there was corporate funding involved. There are definitely potential problems waiting to happen related to this issue.

For the papers previously published, the reviewers were quick to point out these issues when they did feel that a conflict had interfered with the presentation of the paper. In some cases, I have sent submissions to the Board for their evaluation before decided to even consider a paper for publication. The Angle Orthodontist does not have a rigid policy to prohibit corporate funding or even investment or ownership of a company whose product was used in a study. On the other hand, we generally do not publish overt product-testing papers.

Recently, there was a paper submitted testing and comparing two different customized bracket systems to a generic system. One of the customized bracket systems came out on top and the owner of the company happened to be one of the authors. Since there was no real scientific reason or hypothesis as to why that bracket would perform better, we decided not to publish the paper. I think, if there was a valid hypothesis proposed, the data supported it, and the wording was reasonable and not sensational, the paper could have been published with the disclosure accompanying it.

## Recently, it is noted a great increase of the number of articles submitted to the Angle Journal by Brazilian authors. In contrast, the number of accepted articles is smaller when compared to the articles submitted by North American’s authors. May you please cite the greatest difficulties faced by Brazilian authors in their articles and also, how could them increase their chances to achieve success in their submission process to the The Angle Orthodontist? David Normando

It is true that there has been a dramatic increase over the past 10 years in the number of submissions to The Angle Orthodontist from Brazilian authors. As a matter of fact, in the 5 years, 2012-2016, more Angle submissions (768) came from Brazil than any other country in the world. During that same time period, Brazil had the third most articles ACCEPTED for publication, 73, compared to 74 from Turkey, and 115 from the US. In terms of numbers of publications, then, there is not too much of a difference. However, the percent of accepted papers of total papers submitted is somewhat lower for Brazil than the average acceptance rate of 16% over that same time span.

Before I discuss the possible reasons for papers being rejected, I would like to explain that the acceptance rate for papers overall has dropped dramatically since 2000 when Bob Isaacson first became Editor of the journal. In 2001, there were 120 papers submitted and roughly that same number published. By 2012, the number of submissions rose to nearly 1000 per year and it has stayed steady since then. Because the journal is free online and access is open, there is not a potential for expanding the number of published articles because of economic limitations. Therefore, the percent acceptance rate has dropped accordingly to an average of only 14% in 2015. The acceptance rate for papers submitted from the US dropped from 55% in 2010 to 36% in 2015.

There are several factors that figure into why papers submitted to the journal are accepted or rejected. Papers are sent out to reviewers who are chosen primarily because of their expertise in a particular field related to the submitted paper and recent publications on that topic. For the most part, I base my decision solely on their recommendations and the priority they assign, trying to keep the number of acceptances about equal to the number of articles we publish so we don’t fall further behind in our publication backlog, which is roughly 9 months to a year at this point.

I think it is appropriate that the type of articles published in the journal changes as the specialty’s focus and interest changes. We prefer to publish papers on topics that are of most interest to the readers. Currently, the specialty is interested in accelerated orthodontics, aligner therapy, digital technology, CBCT, TADs, airway and sleep apnea, social media, oral health related quality of life, non-compliance treatments, and genetics, among others. There is always a demand for traditional, but controversial, topics such as root resorption, Class II and Class III treatment, surgery, periodontal and bone effects, impactions, and pain. On the other hand, there is less interest than previously on topics such as general morphologic evaluations, cephalometrics, dental development, and materials, especially bonding studies. Generally, we publish less retrospective studies, case reports, case series, and more prospective randomized trials. Several years ago, we stopped publishing general review articles and considered only systematic reviews in their place. Over time, systematic reviews have become more and more narrowly focused so it is sometimes difficult to get those published as well.

One of the common reasons for papers from any specific location to be rejected comes when they are focused on a local population and, therefore, might be of little interest to an international audience. For example, a cephalometric comparison of two ethnic populations in China would probably not be that relevant to readers in Brazil. I get a submission regarding using dental development to assess growth potential in a specific local population almost every week.

When reviewers are solicited, they are given instructions to focus on the scientific content and value of a paper rather than evaluating the English sentence structure and grammar. However, there is no doubt that reading a poorly written paper biases their outlook and may decrease the priority score they assign. In this sense, authors from English speaking countries are at an advantage. Sometimes, it is difficult to discern what an author is trying to say and I have even read sentences in papers where I am sure that the author meant to say the opposite of what is actually written. Even typographical errors, failure to follow the standard structure requirements, or mislabeling figures or tables will lead to questions and cause a reviewer to recommend rejection. In addition, there are often discrepancies between the data presented in the paper itself compared to what appears in the abstract. More commonly, the conclusions of a paper are not directly related to the stated objectives. Some of these are simple cases of losing focus during the writing process. 

One last comment I have is that it is common in many locations around the world for orthodontic programs to require their students to submit a paper to an orthodontic journal as a graduation requirement. Oftentimes, it appears that this is done without appropriate attention from the supervising faculty member. If the student is relatively inexperienced as an author, the submission is focused more like a thesis presentation than a scientific article and may be inappropriate for publication. In all cases, someone in a more senior position should read and edit these papers before they are submitted.

## How do you envision the future of our specialty with the new technologies available and a new generation of orthodontists entering the work force? Bhavna Shroff

I think you can see a clear difference now in the way orthodontics is practiced compared to 20 years ago. When you think about it, the change reflects some of the same change that we have seen in society overall. With the dramatic improvements in technology, we have a generation that is used to having everything at their fingertips instantly so it is not a surprise that orthodontics has shifted in that direction also. Years ago, we used to spend time trying to convince the residents to assess how long a patient was in treatment and not to drag treatment on and on just to attain a perfect result in all cases. They were making treatment last too long and putting the patient at increased risk of iatrogenic harm including more root resorption, white spots, and caries. Also, having patients in treatment over the expected time is bad for practice because the patient finishes paying their contract and you are essentially seeing them without getting paid for it.

Now, on the other hand, I see residents looking at a compromised outcome and saying to me we should remove the braces from the patient without getting a perfect result because the patient doesn’t come to appointments regularly and doesn’t brush their teeth well. Now, I feel like I need to shift my focus on making residents see the value of achieving a more perfect result, to be patient and allow time for the teeth to move properly, and to spend time talking and communicating with patients to achieve their cooperation in a unified effort. It is the opposite of how it was before.

So, it is definitely a combination of new technology and the new generation that goes with it that are fueling changes in the practice of orthodontics. Treatment must be faster, easier for the orthodontist, and less burdensome for the patient. It seems there are limits about how far each of these aspects can progress. Also, it seems we are going to push them all as far as possible.

## In your opinion, how can we best maintain the highest standards of care in orthodontics? Does it come from education in school or board certification soon after graduation? Bhavna Shroff

Maintaining high standards of care is important for retaining the public’s respect for what we do as professionals. I may be mistaken, but I feel that if we let our standards of excellence drop in order to fulfil the public’s demand for fast and cheap service, then, eventually, this will come back and damage our specialty as a whole. How can we claim to be the experts and provide service and outcome that is superior to the generalist if we are actually not delivering on that promise? So, the question is how can we do this?

It has to be a consistent effort and campaign to keep our standards high. We have to send clear, ethical messages right from the start of the recruitment process for new residents when they are graduating from dental school. I always try to emphasize during the interview process for new orthodontic residents, that ethical decisions and ethical logic drive the way our orthodontic program is organized and run. We have to follow through during the education that we provide and reinforce this message. As I said in the answer to a previous question, students used to strive for perfection so hard that it was overdone and actually could lead to harm for the patient. Now, I feel like we need to adjust in the opposite direction. Even if the patients perceive they are done and are anxious to compromise on the outcome and get their treatment done early because the front teeth are straight, the resident has to be taught to sit down and explain to the patient what steps can be taken to make the outcome better if that is the right thing to do.

Then, after graduation, the dental society and orthodontic associations need to follow up with this theme. At least in the US, there is a lot of self-regulation that goes on in professional circles. Professions are given some latitude to set their own standards to some extent. When they fail to do that, there is more chance that the government will come in a set those for them. We have seen some development of formal standards of care from our professional societies. I think, to some extent, this is part of an attempt to begin controlling this. I am sure we will see this issue evolve in the future and I think the professional societies will be leading the charge.

## Could you please comment on the medical necessity of orthodontics? Bhavna Shroff

I have to say that I was never, even when I entered orthodontics as a student, under the impression that orthodontic treatment, in general, was medically necessary. It was always clear to me that malocclusion is not a fatal disease. I was also aware that the evidence did not support any benefits for performing orthodontics toward improving risk of periodontal damage, caries development, or TMJ disturbances. I have always had a great respect for clear scientific results. Also, I was always comfortable myself and accepted that patients generally seek care for esthetic reasons. Therefore, I had no problem explaining to patients and parents that the benefits, clearly, were esthetic. The biggest and most common benefit that we provide as orthodontists is making our patients look better. Deep down, I think we always knew that.

It is only recently that we have accumulated research results documenting the benefits that looking better can actually provide to patients. I feel pretty confident explaining to patients that there is evidence supporting that having a better smile will improve their social lives and help them make a good impression overall. In the long term, this could actually lead to a better job and more successful career. Over the time of my career, as orthodontic care has become more efficient and actually become more affordable for a larger segment of the population, it has been satisfying to see a greater number of people and more diverse portion of society benefit from these changes.

I think the other side of this issue is using the term “medical necessity” as a qualifier for health insurance coverage or for coverage under a government-sponsored health plan. At least in my state within the US, Virginia, the government health system will cover the cost of orthodontic treatment for underprivileged children who qualify by meeting certain conditions. Often these “conditions” are qualifiers for “medical necessity” - such as impacted teeth, gingival impingement, crossbite, severe overjet, etc, or a certain severity of crowding or interarch discrepancy -, which are calculated using a point system. For many years now, this system has enabled many underprivileged children in Virginia to derive social benefits from having orthodontic care.

The flip side of the “medical necessity” issue in the US is the fear by practitioners that insurance companies will start adopting standards of measurement similar to a government sponsored plan, to decide if they will provide orthodontic coverage for their subscribers. This has already happened to some extent in certain parts of the US. This is, in a way, also a political issue. If practitioners need to prove “medical necessity” in order for their patients to benefit from the insurance coverage provided to them by their employers, then it will create a lot of paperwork, add a lot of bureaucratic holdups, and probably increase costs. As you know, the political system in the US was recently flipped upside down, possibly partly as a rebellion against this level of bureaucracy, so it will be interesting to watch how the scenario plays out.

## What are your thoughts on general dentists practicing orthodontics? Bhavna Shroff

I think that general dentists can and should perform orthodontic treatment or procedures within their practice to the extent to which they are knowledgeable to do so. Just as they can perform endodontic or surgical procedures as part of their scope of practice, they need to understand their limitations and not take on cases that are outside of their management abilities. For the most part, this should be interceptive procedures in children, alignment of teeth in non-growing patients, and limited tooth movement to facilitate other dental procedures they might plan to perform. This is purely a generalization because I have known general practitioners at both extremes: those that have exceptional interest and have accumulated enough knowledge and experience to treat complex cases, as well as those who fail to recognize when even a minor interceptive action, such as extracting a primary tooth when clearly indicated, might be of significant benefit to avoid development of a major occlusal problem. No matter the extent to which they choose to perform orthodontics in their practice, general dentists must be, and are, held to the same standard of care as specialists.

Part of the problem is that there is not enough time or focus available as part of the normal dental curriculum to educate generalists adequately about orthodontic diagnosis and treatment. Some of this is just a matter of practicality. Whereas most of dentistry revolves around performing single visit procedures, orthodontic treatment usually goes on for months or even years, requiring multiple visits, and the dental curriculum is too busy to accommodate this difference. Providing time in the schedule for dental students to see the same patient every 4 to 6 weeks over the course of a year or more with the same supervising faculty member for continuity, creates logistical problems within the massive scope of everything else they need to be doing. Also, there are not enough orthodontic faculty members hired at most schools to accomplish this because the schools don’t consider it a priority. The resources go to other disciplines. Honestly, I think most dental students are distracted by the other procedural disciplines during dental school and have little interest in orthodontics until they get out of school and realize then how orthodontics could fit into their practice.

Overall, I don’t really see general dentists providing orthodontic care as a big threat to the specialty of orthodontics in the long term. Orthodontists are more knowledgeable, better organized, more experienced, and more efficient at providing orthodontic care than general dentists. General dentists are busy doing other things and usually can’t reorganize their whole practice to provide the same efficient orthodontic care as specialists. If the specialty can do a good job transmitting that message to the public, and hold up their part by delivering superior care, I think the balance will be in favor of orthodontists.

## You have expended your entire career-life teaching in an academic setting. How has orthodontic teaching evolved during the last three decades? Jorge Faber

Orthodontic teaching has probably changed less than I perceive over the past 30 years, which is the time frame you asked me to consider. I went to school at the University of Connecticut and began my orthodontic education precisely 30 years ago. At that time, the program there was organized as I would envision any true graduate program would be in any learned discipline. That is, it was organized as small group discussion seminars where a Professor would spend time with the small group of students enrolled, discussing a topic related to orthodontics. Rather than lecturing to the group, there would be reading assignments and the graduate students would present what they read to the group and then the group would discuss the topic based on what was known and what was not yet known. The Professor would then prompt the students to think in different ways and a learned discussion would ensue. This might result in more unanswered questions that could be considered as future topics for research.

Despite my experience at the University of Connecticut, I believe that in many programs orthodontics continues to be taught in a more outdated, traditional way. That is, there is an expert in the specialty, whether it be a full-time or part-time faculty member or even a guest speaker, who comes in and shares their knowledge in the form of a lecture with the graduate students. There may indeed be an opportunity for questions and discussion but, ultimately, it is the opinion or interpretation given by the Professor that is regarded as the knowledge transmitted. I believe this type of education is fine for the undergraduate level but, at higher levels such as specialization, the students should assume more responsibility in the learning process.

Furthermore, I think it is essential, in this era of “evidence-based” clinical knowledge and practice, that graduate programs be focused on the current literature and scientific evidence that we have in our field. I hope that this is indeed the case at all orthodontic programs but I am doubtful that this is true. It is much more difficult and time-intensive to structure the learning process around current literature than it is to deliver a standard series of lectures to the students on an annual basis.

To answer the original question directly, I would surmise that more programs now are structured in group discussion format than they were 30 years ago. I think there is less reliance and more skepticism on the part of the students for believing without question what they hear in lectures. Also, I think graduate students today get more hands-on clinical experience overall than they did 30 years ago and that is very valuable in many ways. Most obviously, hands-on experience makes what students learn in lecture and seminar more relevant to clinical practice and it helps them incorporate that knowledge more universally.

As an aside, my opinion is that there was more emphasis on technical perfection 30 years ago: more wire bending and precision of detail. Now, despite the more highly defined clinical assessments that have come from the board examination processes (ABO standards, for example), I feel like students are more focused on efficiency of treatment, providing an adequate result (rather than achieving perfection), and patient satisfaction, than they are on achieving final occlusal excellence.

## What is/are the advice(s) that you would give to young enthusiastic orthodontic teachers at the early days of their careers? Jorge Faber

For new faculty members (teachers), they are certainly entering a different world than I entered 30 years ago. Overall, the model has not changed that much over the years. Faculty members in most places must supplement their income by seeing patients privately within the university or outside in private practice. As previously, most faculty members go through periods of struggle where they question whether they want to, or can afford to, remain in academics or whether they should leave and go into private practice full-time where the monetary rewards will be greater.

I would advise them that an academic career can be very rewarding in many ways. Most obviously, there is the reward that you get directly by working with young students who look up to you and you share your knowledge with them and guide them along their career pathways. But, for those who have a deeper calling, I think there is an inner satisfaction gained by intellectual challenge and trying to answer questions that the specialty struggles with from a scientific point of view. I don’t think that an academic career is the answer for someone who is uncertain or lacks the confidence to pursue private practice or for someone who is just not ready to commit to a definite career path.

For me, one of the biggest advantages of pursuing an academic career was being able to have a greater variety of activities in my life, rather than just treating patients every day. I spend time in seminar with students, supervising their work in clinic, lecturing to dental students, working with colleagues in other departments, and helping students and orthodontic residents with their research projects. Also, I see patients of my own a half-day per week. It would be hard for me to give up the diversity I have in my job to do any one of those things all the time.

For someone starting out in an academic career today, the biggest challenge is to fulfill the academic requirements for advancement and promotion. In the US, the AAO Foundation has many great opportunities for new faculty to supplement their incomes a little bit and also give them funding for research. This provides a great start. It is enormously important to have a mentor, preferably at the same school, who will guide and help you through the research development, analysis, and publication phases. On the other hand, a new faculty has to be self-motivated and work well independently. They shouldn’t expect that a mentor will be writing research proposals or publications for them.

Besides research funding, publication is the major way institutions evaluate the success of academic faculty. Compared to many years ago, it is much more difficult to have papers accepted in major orthodontic journals today. For example, in 2000, The Angle Orthodontist received about 200 submissions and published about 125 articles. Today, the journal receives about 1000 submissions a year but still publishes about 135.

The last bit of advice I would give to potential and new faculty members in orthodontics is to stick with it if you are feeling doubtful. There were many times that I questioned whether it was worth all the extra work and stress and whether I could continue to work long hours for less money than many of my private practice colleagues. I thought about leaving for private practice many times. In a way, just knowing that I COULD leave for private practice at any time was what got me through the hard times. Now, looking back, I can not imagine a career path that could have been more rewarding in so many intangible ways.

## Based on your brilliant carrier in orthodontics, what is the most important piece of advice you would give to residents in orthodontics around the world? Andre W. Machado

The advice I would give residents all over the world is the same in a way to that I would give all practitioners of orthodontics (and actually, of any profession) all over the world. It is important to realize that things do not remain static. What you learn is school is the current state of your profession, practice, or occupation. Hopefully, you learn basic principles that will remain applicable even as the state of what is known and practiced will change over time. You have to be knowledgeable in what changes occur in your profession and specialty. You have to be willing to change as new knowledge is accumulated in your field.

In orthodontics, more than many other aspects of dentistry, changes occur continuously. There are many orthodontists who are excited and motivated to try new techniques and apply new technology as it becomes available. In the current environment, technology especially changes rapidly. Superimposed on this is the introduction, and re-introduction, of new and old theories of how orthodontists think that diagnosis and treatment should be conducted. Therefore, old theories get re-introduced as new theories so it is important to understand the history of the specialty and know that there is no magic solution to the problems we encounter with our patients every day. On the other hand, it is important to not be closed-minded and afraid to explore new ideas that may improve the way we practice.

In other words, new residents should not expect that the practice of orthodontics, as they learn it, will continue indefinitely into the future. They should be open to changing the way they practice. They should be “life-long learners”. But, also, they should not be too quick to abandon the basic principles that have been scientifically proven in the past. It is a delicate balance between past knowledge and new knowledge. You have to be confident that, in the long run, things will always be getting better and more efficient.

